# Clinical Presentation, Aetiology, and Outcomes of Meningitis in a Setting of High HIV and TB Prevalence

**DOI:** 10.1155/2015/423161

**Published:** 2015-09-30

**Authors:** Keneuoe Hycianth Thinyane, Keanole Mofona Motsemme, Varsay Jim Lahai Cooper

**Affiliations:** ^1^Department of Pharmacy, National University of Lesotho, Roma 180, Lesotho; ^2^Department of Internal Medicine, Queen Mamohato Memorial Hospital, Maseru 100, Lesotho

## Abstract

Meningitis causes significant morbidity and mortality globally. The aim of this study was to study the clinical presentation, aetiology, and outcomes of meningitis among adult patients admitted to Queen Mamohato Memorial Hospital in Maseru, Lesotho, with a diagnosis of meningitis. A cross-sectional study was conducted between February and April 2014; data collected included presenting signs and symptoms, laboratory results, and clinical outcomes. Descriptive statistics were used to summarise data; association between variables was analysed using Fisher's exact test. 56 patients were enrolled; the HIV coinfection rate was 79%. The most common presenting symptoms were altered mental status, neck stiffness, headache, and fever. TB meningitis was the most frequent diagnosis (39%), followed by bacterial (27%), viral (18%), and cryptococcal meningitis (16%). In-hospital mortality was 43% with case fatalities of 23%, 40%, 44%, and 90% for TB, bacterial, cryptococcal, and viral meningitis, respectively. Severe renal impairment was significantly associated with mortality. In conclusion, the causes of meningitis in this study reflect the high prevalence of HIV and TB in our setting. Strategies to reduce morbidity and mortality due to meningitis should include improving diagnostic services to facilitate early detection and treatment of meningitis and timely initiation of antiretroviral therapy in HIV-infected patients.

## 1. Introduction

Meningitis is a clinical syndrome characterized by inflammation of the meninges; it is one of the most common infectious diseases of the central nervous system (CNS) [1]. Most cases of meningitis are caused by bacteria or viruses; however fungi and parasites can also cause meningitis, especially in immunocompromised patients [2–4]. Infection with the human immunodeficiency virus (HIV) is emerging as a major risk factor for meningitis in adults [5–7]. Studies show that tuberculous meningitis (TBM) and cryptococcal meningitis (CM) are among the most common CNS opportunistic infections in patients with HIV/AIDS in sub-Saharan Africa and Asia [8–11].

Meningitis causes significant morbidity and mortality globally [3, 9, 12, 13]. Long term sequelae of bacterial meningitis in adults include hearing and visual loss, seizures, and cognitive impairment [14]. Neurological and neuropsychological deficits have also been reported in adults following cryptococcal, tuberculous, and viral meningitis [4, 15, 16]. Mortality from meningitis appears to be much higher in developing countries than in developed countries [4, 5, 17]. Factors that contribute to this high mortality include delay in diagnosis/treatment of meningitis and severe immunosuppression in HIV-infected patients [12, 13].

In Lesotho the prevalence of HIV infection is estimated at 23% of the adult population; the country also has a high incidence of tuberculosis (TB) estimated at 633 per 100 000 of the population [18, 19]. National data show that in 2009 the rate of TB-HIV coinfection among tested patients was 76.5%; 22% of all notified new TB cases during this period were extrapulmonary [19]. In Lesotho as in other countries with a high HIV burden, meningitis is a major cause of hospital admission among HIV-infected adults [8, 19]. At present there are limited data on the causes of meningitis among adults in Lesotho [20, 21]; in addition the influence of HIV infection on the pathogenesis and clinical outcomes of meningitis have not been investigated. The aim of this study was to investigate the clinical presentation, aetiology, and outcome of meningitis among patients admitted to a tertiary level hospital in Maseru, Lesotho.

## 2. Methods

A cross-sectional study was conducted at Queen Mamohato Memorial Hospital (QMMH), the national tertiary referral hospital in Maseru, Lesotho, over a three-month period between February and April 2014. Patients presenting with signs and symptoms suggestive of meningitis, headache, neck stiffness, fever, photophobia, and/or altered mental status, were screened on admission to the medical wards. All patients 18 years and older with an admission diagnosis of meningitis were eligible to participate in the study. Exclusion criteria were discharge or death within 24 hours of admission.

Prior to data collection, written informed consent was obtained from each patient; for patients who were too ill to communicate, permission to enroll the patients in the study was obtained from the next of kin. Data collected at admission included age, gender, and medical history (history of present illness and concurrent diseases including HIV infection). Prehospitalisation medical records were reviewed and history of antiretroviral therapy (ART), anti-TB therapy (ATT), isoniazid preventive therapy (IPT), and use of cotrimoxazole or other antimicrobial agents was noted. Clinical evaluation of patients for meningitis was carried out according to hospital protocols. All patients underwent a physical examination and neurological assessment on admission and when not contraindicated immediate lumbar puncture (LP) was performed prior to the first dose of antibiotics. Initial cerebrospinal fluid (CSF) analysis for all patients included macroscopy, white cell count (WCC) and differential, protein and glucose levels, Gram stain and culture, India ink stain, and CSF cryptococcal antigen test. Repeat LP was performed when necessary; additional investigations included bacterial antigen detection by CSF latex agglutination tests for* Streptococcus pneumoniae*,* Streptococcus* group B,* H. influenzae* B,* N. meningitidis* serogroups A, C, Y, and W-135, and* N. meningitidis* serogroup B/*E. coli* K1 and bacterial culture and antibiotic susceptibility testing. Computed tomography (CT) scanning of the head was performed in some, but not all patients with focal neurological deficits and/or altered mental status prior to lumbar puncture or within 24–48 hours of admission to exclude intracranial space occupying lesions and other causes of raised intracranial pressure. Patients with clinical suspicion of TB meningitis and/or HIV infection underwent sputum acid fast bacilli (AFB) smear and culture testing and chest radiography to screen for the presence of TB. Patients with an unknown or negative HIV status on admission were offered voluntary HIV counselling and testing; consenting patients were tested for HIV infection with a rapid test or HIV ELISA. Routine biochemical investigations for all patients included full blood count, liver function tests, and urea and electrolytes.

Clinical, radiological, microbiological, and other laboratory data were evaluated and the diagnosis of meningitis was made based on a combination of clinical and CSF findings (CSF protein and glucose levels, cell count and differential, microscopy, and culture) as explained below.

### 2.1. Bacterial Meningitis

Bacterial meningitis was diagnosed based on a positive CSF Gram stain and culture or a positive bacterial antigen test (BAT). In patients without a definitive microbiological diagnosis, bacterial meningitis was diagnosed when patients had a compatible clinical presentation (sudden onset of fever, headache, altered mental status, or other meningeal signs) and typical CSF findings (CSF pleocytosis with polymorphonuclear cell predominance, low glucose, and elevated protein). Patient response to antibiotic therapy was closely monitored; patients showing no clinical improvement within 7–10 days of initiation of empiric antibiotic therapy underwent further diagnostic tests.

### 2.2. Cryptococcal Meningitis

A diagnosis of cryptococcal meningitis was made in the presence of a positive CSF India ink stain or CSF cryptococcal antigen (CSF CRAG) test.

### 2.3. TB Meningitis

A diagnosis of TB meningitis was made when patients had clinical features of meningitis with negative CSF Gram stain and cultures for bacteria, negative CSF cryptococcal antigen test, and at least one of the following: (i) characteristic CSF findings (CSF pleocytosis with lymphocytic predominance, low glucose, and elevated protein); (ii) evidence of active tuberculosis at another site (e.g., the lungs); or (iii) brain CT findings suggestive of TM such as basal meningeal enhancement or hydrocephalus. Patients showing resolution of constitutional symptoms within 14–21 days of starting anti-TB treatment were continued on treatment and did not undergo further investigations before discharge. CSF acid-fast bacilli smear or culture studies were not performed.

### 2.4. Viral Meningitis

Viral meningitis was diagnosed when patients had clinical features of meningitis with negative CSF Gram stain and cultures for bacteria, negative CSF cryptococcal antigen test, and typical CSF findings (CSF lymphocytic predominance, normal or slightly elevated protein, and normal glucose) with exclusion of nonviral causes of aseptic meningitis. CSF polymerase chain reaction (PCR) testing for viral pathogens was not done.

All data analyses were performed using SPSS version 20.0. Descriptive statistics were represented as frequencies (%) and median and interquartile range (IQR). Fisher's exact test was conducted to examine the relationship between the dependent variable (in-hospital mortality) and other categorical variables including age, HIV status, CD4 count, and estimated glomerular filtration rate (eGFR) on admission. A *p* value less than 0.05 was considered statistically significant.

The study was approved by the Lesotho Ministry of Health Research and Ethics Committee.

## 3. Results

A total of 72 patients with clinical suspicion of meningitis were enrolled. 16 patients were subsequently excluded; of these 12 patients were diagnosed with other CNS diseases and 4 did not have a clear final diagnosis. Of the 56 patients included in the final analysis, 57% (*n* = 32) were female and the median age was 35 years (IQR: 28–44); 79% (*n* = 44) were HIV positive (Table 1). 26 of the 44 HIV-infected patients (59%) were receiving antiretroviral therapy; among these, 13 patients had been on ART for less than 3 months, 7 patients for 3–6 months, and 6 patients for more than 6 months; 5 patients were taking cotrimoxazole prophylaxis and 1 patient was on IPT. 9 patients (16%) were on antituberculosis treatment on admission. The most common clinical presentation of meningitis was altered mental status followed by neck stiffness and headache; less common symptoms included vomiting (*n* = 12), photophobia (*n* = 5), and seizures (*n* = 4). CD4 cell counts were measured for 66% (29/44) of the patients with HIV infection; the majority of these (24/29) had CD4 cell counts <200 cells/mm^3^. More than half of all patients had one or more clinical and biochemical features consistent with critical illness including severe wasting, inability to walk/talk, serum Na^+^ <130 mmol/L (*n* = 16), eGFR <30 mL/min (*n* = 10), and Hb <8.0 g/dL (*n* = 6).

Table 2 shows CSF findings and clinical outcomes among the 56 study participants: 22 patients (39%) were diagnosed with TB meningitis, 15 (27%) had bacterial meningitis, 10 (18%) had viral meningitis, and 9 (16%) had cryptococcal meningitis. HIV coinfection rates were greater than 70% for all types of meningitis; the proportion of patients with CD4 cell counts below 100 cells/mm^3^ was 100% (9/9) for cryptococcal meningitis, 50% (5/10) for viral meningitis, 33% (5/15) for bacterial meningitis, and 0% (0/22) for TB meningitis. The majority of the patients had CSF parameters, CSF cell, protein, and glucose findings, characteristic of the various types of meningitis. Of the 15 patients diagnosed with bacterial meningitis, 9 had a CSF pleocytosis with a polymorphonuclear cell predominance reported in 7 patients; 5 patients had low CSF glucose, and 11 had elevated protein. Positive Gram stain results were obtained on initial CSF specimen for 9 patients. Six patients were diagnosed on the basis of CSF findings after repeat LP. A positive bacterial antigen test was reported in 4 patients (*S. pneumoniae*, *n* = 3;* H. influenzae*, *n* = 1) and a positive Gram stain in 3; one patient had a positive Gram stain and BAT result. Two patients with microbiologically confirmed bacterial meningitis had a normal CSF study (CSF white cell count up to 5 cells/mm^3^, glucose >2.2 mmol/L, and protein <0.45 g/L). Of the 9 patients diagnosed with cryptococcal meningitis, 6 had a positive CSF India ink stain and 8 were CSF CRAG positive; 5 patients had a CSF white cell count >5 cells/mm^3^ with mononuclear cell predominance in 4 patients; low CSF glucose was found in 5 and elevated protein in 8 patients. One patient had a normal CSF study at diagnosis. 22 CSF Gram stain-, bacterial antigen test-, and CSF CRAG-negative patients were diagnosed with TB meningitis. A CSF pleocytosis, median CSF WCC 217 cells/mm^3^ (IQR: 58–435), was observed in all patients with TBM. 14 patients had CSF white cell counts above 100/mm^3^, and 20 had a CSF lymphocytic predominance (>90% mononuclear cells). The proportion of patients with low CSF glucose and elevated CSF protein was 12/22 and 20/22, respectively. CSF analysis in patients with viral meningitis showed acellular CSF (*n* = 8) or a mild lymphocytic pleocytosis (CSF white cell count <30 cells/mm^3^; *n* = 2), normal CSF glucose levels (*n* = 9), and elevated protein (*n* = 7).

Empirical antimicrobial therapy was started with intravenous ceftriaxone; 52% of the patients received adjunctive dexamethasone on admission. Patients diagnosed with TBM were started on a regimen of isoniazid, rifampicin, ethambutol, and pyrazinamide; bacterial meningitis was treated with ceftriaxone, cryptococcal meningitis with amphotericin B, and viral meningitis with acyclovir. Supportive therapy included analgesics/antipyretics and antiemetics. Patients were closely monitored and in cases of clinical deterioration, further investigations were performed and change in therapy was instituted as necessary. The median hospital stay for discharged patients was 11 days, range 6–25 days. The overall in-hospital mortality was 43% (24/56) with more than half of the deaths (14/24, 58%) occurring during the first 7 days of hospital admission. The case fatality was 90% for viral meningitis, around 40% each for bacterial and cryptococcal meningitis and 23% for TB meningitis. In-hospital mortality was higher although not statistically significant among older patients, 67% (4/6) versus 40% (20/50), patients with HIV infection, 48% (21/44) versus 29% (2/7), and those with altered mental status, 50% (21/42) versus 21% (3/14) (Table 3). Severe renal impairment (eGFR < 30 mL/min) was significantly associated with in-hospital mortality, *p* = 0.008 (Fisher's exact test).

## 4. Discussion

We investigated the clinical presentation, aetiology, and outcomes of meningitis among adult patients admitted to a tertiary level hospital in Maseru, Lesotho. TB meningitis was the most common cause of meningitis (39%) followed by bacterial meningitis (27%), viral meningitis (18%), and cryptococcal meningitis (16%). The percentage of HIV infection was 79%, with the majority of the patients presenting with symptoms and signs of advanced HIV infection (stage III/IV events) on admission. Studies from sub-Saharan Africa show that HIV coinfection is common among adults with meningitis [7, 8, 22]. In a study conducted in South Africa, Jarvis et al. [6] found HIV coinfection rates above 90% among patients diagnosed with bacterial, cryptococcal, and tuberculous meningitis. The immunodeficiency caused by HIV predisposes individuals to opportunistic infections by pathogens such as* Cryptococcus neoformans* and* Mycobacterium tuberculosis* which typically do not cause CNS infections in immunocompetent hosts [23]. Research indicates that the spectrum of meningitis varies between countries: in parts of Africa and South East Asia, cryptococcal meningitis is the leading cause of adult meningitis, with most of the cases occurring in patients with CD4 counts <100 cells/mm^3^ [10, 24, 25]. In our study, TB meningitis was the most common form of meningitis among adults, a finding which is consistent with data from other studies conducted in Southern Africa [5, 22]. Lesotho, like South Africa, has a high HIV and TB burden with HIV/TB coinfection rates above 70%. In this study, 59% of all patients (*n* = 33) were treated with anti-TB drugs during hospitalisation; of these two-thirds (*n* = 22) had been diagnosed with TB meningitis and a third had other forms of TB including pulmonary tuberculosis. Patients with HIV infection and active TB are at an increased risk of extrapulmonary tuberculosis [26]; in these patients TB meningitis is caused by the hematogenous dissemination of the tubercle bacilli from the primary site of infection such as the lungs. Research indicates that starting antiretroviral therapy early in the course of HIV infection and the use of prophylactic antimicrobial regimens might help reduce the incidence of CNS opportunistic infections in HIV-infected patients [10].

88% of the patients presented with at least two of the four signs and symptoms of fever, headache, neck stiffness, and altered mental status. Studies show that about 20–70% of adults with meningitis present with varying degrees of altered mental status ranging from confusion to impaired level of consciousness [27–29]. Multiple studies have shown that neurological signs at presentation are prognostic of poor clinical outcomes in patients with meningitis. In this study, mortality was higher though not statistically significant among patients with altered mental status. In a recent study, Jarvis et al. [12] found that altered mental status was an independent predictor of acute mortality in patients with cryptococcal meningitis. Similar findings have been reported for patients diagnosed with bacterial meningitis [27] and TB meningitis [22]. In general CSF cell and biochemistry findings in our study were typical of the different forms of meningitis; however CSF white cell counts were lower than those reported in adult references. More than a third of all patients with microbiologically confirmed bacterial and cryptococcal meningitis had atypical CSF findings: 6 of the 15 patients with bacterial meningitis had CSF white cell counts ≤5/mm^3^ and 2 had a normal CSF study; the proportions were 4/9 and 1/9, respectively, among patients with cryptococcal meningitis. These findings are similar to those reported among populations with (predominantly) HIV-associated meningitis [6, 13, 22]. Abnormal CSF cell and biochemistry findings may cause diagnostic uncertainty especially in the presence of negative CSF microbiology results. The diagnosis of meningitis remains difficult, particularly in resource-limited settings. In diagnosing TB meningitis, CSF AFB smear has relatively low sensitivity, reported to be less than 10% in several studies [4, 13, 22], and although CSF AFB culture has greater sensitivity, it can take up to 8 weeks to obtain results, leading to a delay in diagnosis and initiation of anti-TB therapy. For these reasons ancillary investigations including neuroimaging studies and chest radiography are often necessary to confirm/rule out TB meningitis. Confirmatory diagnostic tests for viral meningitis include CSF PCR for nucleic acids and CSF viral culture; however these tests are not widely available. There is a need for sensitive, rapid laboratory tests to enable prompt diagnosis of meningitis and initiation of appropriate antimicrobial therapy.

The overall mortality was 43%; severe renal impairment (eGFR < 30 mL/min) was the only variable significantly associated with in-hospital mortality among the study participants. Some studies indicate that a positive HIV status is predictive of mortality in adults with meningitis [8, 26, 28]. In this study, the mortality rate was higher though not statistically significant among HIV-positive patients compared to those who were HIV negative. Notably, all of the patients with severe renal impairment (*n* = 10) were also HIV-infected; 6 of these patients were receiving antiretroviral therapy (tenofovir/lamivudine/efavirenz) on admission and 4 were ART naive. In the absence of other comorbidities, it is highly likely that renal impairment in these patients was HIV related; either HIV-associated nephropathy or tenofovir induced renal failure. In this study, the case fatality rate for bacterial and cryptococcal meningitis was 40% and 44%, respectively, which is similar to that reported elsewhere [5, 9, 27]. Despite advances in diagnosis and treatment, mortality from bacterial and cryptococcal meningitis remains high, especially in developing countries [12, 17]. The case fatality for TBM was 23%, which is lower than that reported in other studies [13, 22]. A possible explanation for this difference is that although the majority of the TB meningitis cases in this study were also HIV-associated, the median CD4 count among HIV-infected TBM patients (119 cells/mm^3^, IQR: 100–277) was higher than that reported in the two studies above. Severe immunosuppression is significantly associated with increased in-hospital mortality in patients with TB meningitis [22]. We found a case fatality rate of 90% for patients with viral meningitis; all of the patients with viral meningitis in this study were HIV-infected. In immunocompetent adults, viral meningitis is often a benign, self-limiting condition that resolves spontaneously without any sequelae; however in immunocompromised patients, viral meningitis is frequently associated with a significant mortality [30]. Approximately 10% of HIV-infected patients develop HIV meningitis which occurs primarily at seroconversion; other aetiologic agents in HIV-associated viral meningitis include Epstein-Barr virus (EBV), herpes simplex virus (HSV) types 1 and 2, and cytomegalovirus [30, 31]. Limited data exists regarding the aetiology of viral meningitis in sub-Saharan Africa. In two recent studies conducted in Malawi [31] and Uganda [32], EBV was identified as the most common cause of viral meningitis among HIV-infected patients while HSV infection was rarely found. In our setting, viral PCR testing is not available to confirm the diagnosis of viral meningitis and guide antiviral therapy. Possible consequences of this may include misdiagnosis of patients with atypical CSF findings, inappropriate antimicrobial therapy, or a delay in starting appropriate antimicrobial therapy. In this study, all patients with suspected viral meningitis were treated with acyclovir. Acyclovir has been used successfully to treat HSV meningitis; however the treatment of viral meningitis remains challenging as there are often no definitive effective therapies for most pathogens including EBV [30].

This study has several limitations. First of all, although this was a cross-sectional study, we did not obtain a detailed medical history of the type and duration of symptoms and/or recent use of antimicrobial agents for some of the study participants; in addition some investigations such as CD4 counts were not done routinely thus limiting the use of such variables in further data analyses. Secondly, the limited range of diagnostic tests performed, low sensitivity of the available laboratory techniques, and the presence of atypical clinical and laboratory findings complicate the diagnosis of meningitis; this could have led to exclusion of some cases and misclassification of others. Finally, the study sample was small which limited statistical analysis. Despite these limitations, this study provides evidence of the association between HIV infection and meningitis and the relatively higher prevalence of TB meningitis in our setting and further highlights the difficulties in diagnosing meningitis in a resource-limited setting. In the current study, the primary outcome of interest was death or survival to hospital discharge; future research should include neuropsychological evaluation of patients to assess disability at discharge. In addition, long term follow-up studies are required to study the long term sequelae of meningitis and the incidence of meningitis recurrence especially in patients with HIV infection.

## 5. Conclusions

In conclusion, the majority of the meningitis cases in this study were HIV-associated. TB meningitis was the most common cause of meningitis followed by bacterial, viral, and cryptococcal meningitis. In-hospital mortality was high with case fatality rates of 23%, 40%, 44%, and 90% for TB, bacterial, cryptococcal, and viral meningitis, respectively. There is a need for sensitive, rapid, and affordable laboratory tests to enable prompt diagnosis and treatment of meningitis. Early diagnosis of HIV infection and timely initiation could reduce morbidity and mortality from HIV-associated meningitis in this setting.

## Figures and Tables

**Table 1:** Demographic and baseline characteristics of the study participants.

Variable	*n* (%)
Female gender, *N* = 56	32 (57)
Age group, years, *N* = 56	
18–24	8 (14)
25–34	20 (36)
35–49	22 (39)
>49	6 (11)
HIV status, *N* = 56	
Positive	44 (79)
Negative	7 (13)
Unknown	5 (9)
% on antiretroviral therapy, *N* = 44	26 (59)
Clinical presentation, *N* = 56	
Altered mental status	42 (75)
Neck stiffness	38 (68)
Headache	30 (54)
Fever	24 (43)
Seizures	4 (7)
Baseline biochemistry, median (IQR)	
CD4 count, cells/mm^3^, *N* = 29	100 (53–155)
Na^+^, mmol/L, *N* = 46	131.0 (128.0–137.3)
K^+^, mmol/L, *N* = 48	4.0 (3.4–4.9)
eGFR, mL/min, *N* = 46	57 (26–78)
ALT, U/L, *N* = 42	39 (27–77)
Hb, g/dL, *N* = 50	11.0 (9.0–13.0)
PLT, ×10^3^/L, *N* = 48	273 (186–336)

Data presented as *n* (%) unless otherwise stated; ALT: alanine transaminase; Hb: haemoglobin; *n*: number of patients; *N*: total number of patients for whom the analysis was performed; PLT: platelets.

**Table 2:** Cerebrospinal fluid analysis and clinical outcomes.

	BM	CM	TBM	VM
	(*n* = 15)	(*n* = 9)	(*n* = 22)	(*n* = 10)
HIV positive, *n*/*N* (%)^a^	11/13 (85)	8/9 (89)	15/19 (79)	10/10 (100)
% on ART^b^	8/11 (73)	3/8 (38)	11/15 (73)	4/10 (40)
CD4 count, median, cells/mm^3^ (IQR)	162 (91–257)	18 (12–41)	119 (100–277)	102 (75–129)

CSF analysis				
Gram stain (+)	12^c^/15 (80)	0 (0)	0 (0)	0 (0)
Bacterial antigen test (+)	4^c^/15 (27)	0 (0)	0 (0)	0 (0)
India ink (+)	0 (0)	6/9 (67)	0 (0)	0 (0)
CSF CRAG (+)	0 (0)	8/9 (89)	0 (0)	0 (0)
White blood cells				
Range, cells/mm^3^	0–420	0–184	8–2393	0–18
Median, cells/mm^3^ (IQR)	7 (2–147)	10 (0–86)	217 (58–435)	0 (0-0)
>5 cells/mm^3^, *n*/*N* (%)	9/15 (60)	5/9 (56)	22/22 (100)	2/10 (20)
Lymphocytic pleocytosis^d^	2/9 (22)	4/5 (80)	20/22 (91)	2/2 (100)
Glucose				
Median, mmol/L (IQR)	3.0 (1.1–3.8)	2.0 (1.0–2.9)	1.1 (0.6–2.3)	3.0 (2.7–3.6)
<2.2 mmol/L, *n*/*N* (%)	5/15 (33)	5/9 (56)	14/22 (64)	1/10 (10)
Protein				
Median, g/L (IQR)	0.6 (0.4–1.5)	1.0 (0.7–1.8)	2.0 (1.7–4.4)	0.5 (0.4–0.7)
>0.45 g/L, *n*/*N* (%)	11/15 (73)	8/9 (89)	20/22 (91)	7/10 (70)

Hospital stay, median days (IQR)	11 (9–12)	17 (17–23)	11 (8–11)	23 (23-23)
Mortality rate, *n*/*N* (%)	6/24 (25)	4/24 (17)	5/24 (21)	9/24 (38)
Fatality rate, *n*/*N* (%)	6/15 (40)	4/9 (44)	5/22 (23)	9/10 (90)

^a^HIV infection rate expressed as a percentage of patients with known HIV status; ^b^expressed as a percentage of patients with HIV infection; ^c^positive Gram stain and bacterial antigen results reported for initial and repeat CSF specimens; ^d^calculated as a percentage of patients with CSF white cells >5 cells/mm^3^; BM: bacterial meningitis; CM: cryptococcal meningitis; MN: mononuclear cells; *n*: number of patients; *N*: total number of patients for whom the analysis was performed; PMN: polymorphonuclear cells; TBM: tuberculous meningitis; VM: viral meningitis.

**Table 3:** Analysis of factors associated with in-hospital mortality.

	Survived	Died	*p* value (Fisher's exact test)
Age, years			
>49	2	4	0.385
≤49	30	20	
Gender			
Male	14	10	1.000
Female	18	14	
HIV status			
Positive	23	21	0.436
Negative	5	2	
CD4 count, cells/mm^3^			
<100	7	7	0.715
≥100	9	6	
Altered mental status			
Yes	21	21	0.072
No	11	3	
Seizures			
Yes	3	1	0.627
No	29	23	
Serum sodium, mmol/L			
<130	11	5	0.750
≥130	18	12	
eGFR, mL/min			
<30	2	8	0.008
≥30	26	10	

## References

[1] Porter R. S., Kaplan J. L. (2011). *The Merck Manual of Diagnosis and Therapy*.

[2] Bamberger D. M. (2010). Diagnosis, initial management, and prevention of meningitis. *American Family Physician*.

[3] Pyrgos V., Seitz A. E., Steiner C. A., Prevots D. R., Williamson P. R. (2013). Epidemiology of cryptococcal meningitis in the US. *PLoS ONE*.

[4] Christensen A.-S. H., Andersen Å. B., Thomsen V. T., Andersen P. H., Johansen I. S. (2011). Tuberculous meningitis in Denmark: a review of 50 cases. *BMC Infectious Diseases*.

[5] Bergemann A., Karstaedt A. S. (1996). Spectrum of meningitis. *The Quarterly Journal of Medicine*.

[6] Jarvis J. N., Meintjes G., Williams A., Brown Y., Crede T., Harrison T. S. (2010). Adult meningitis in a setting of high HIV and TB prevalence: findings from 4961 suspected cases. *BMC Infectious Diseases*.

[7] Hakim J. G., Gangaidzo I. T., Heyderman R. S. (2000). Impact of HIV infection on meningitis in Harare, Zimbabwe: a prospective study of 406 predominantly adult patients. *AIDS*.

[8] Békondi C., Bernede C., Passone N. (2006). Primary and opportunistic pathogens associated with meningitis in adults in Bangui, Central African Republic, in relation to human immunodeficiency virus serostatus. *International Journal of Infectious Diseases*.

[9] Berhe T., Melkamu Y., Amare A. (2012). The pattern and predictors of mortality of HIV/AIDS patients with neurologic manifestation in Ethiopia: a retrospective study. *AIDS Research and Therapy*.

[10] Tan I. L., Smith B. R., von Geldern G., Mateen F. J., McArthur J. C. (2012). HIV-associated opportunistic infections of the CNS. *The Lancet Neurology*.

[11] Sharma S. K., Kadhiravan T., Banga A., Goyal T., Bhatia I., Saha P. K. (2004). Spectrum of clinical disease in a series of 135 hospitalised HIV-infected patients from north India. *BMC Infectious Diseases*.

[12] Jarvis J. N., Bicanic T., Loyse A. (2014). Determinants of mortality in a combined cohort of 501 patients with HIV-associated cryptococcal meningitis: Implications for improving outcomes. *Clinical Infectious Diseases*.

[13] Luma H. N., Tchaleu B. C. N., Ngahane B. H. M. (2013). Tuberculous meningitis: presentation, diagnosis and outcome in HIV-infected patients at the Douala General Hospital, Cameroon: a cross sectional study. *AIDS Research and Therapy*.

[14] Hoffman O., Weber J. R. (2009). Pathophysiology and treatment of bacterial meningitis. *Therapeutic Advances in Neurological Disorders*.

[15] Schmidt H., Heimann B., Djukic M. (2006). Neuropsychological sequelae of bacterial and viral meningitis. *Brain*.

[16] Lan S.-H., Chang W.-N., Lu C.-H., Lui C.-C., Chang H.-W. (2001). Cerebral infarction in chronic meningitis: a comparison of tuberculous meningitis and cryptococcal meningitis. *QJM: Monthly Journal of the Association of Physicians*.

[17] Gordon S. B., Walsh A. L., Chaponda M. (2000). Bacterial meningitis in Malawian adults: pneumococcal disease is common, severe, and seasonal. *Clinical Infectious Diseases*.

[18] (2009). *Lesotho Demographic and Health Survey*.

[19] Ministry of Health and Social Welfare (MOHSW) (2010). *Annual Joint Review Report 2009/10 FY*.

[20] Thinyane K. H., Cooper V. (2013). Clinical profiles of HIV-infected, HAART-naive patients admitted to a tertiary level hospital in Maseru, Lesotho. *The Internet Journal of Infectious Diseases*.

[21] Thinyane K. H., Cooper V. L. J. (2013). Causes of hospital admission among patients receiving highly active antiretroviral therapy. *Research Journal of Medical Sciences*.

[22] Marais S., Pepper D. J., Schutz C., Wilkinson R. J., Meintjes G. (2011). Presentation and outcome of tuberculous meningitis in a high HIV prevalence setting. *PLoS ONE*.

[23] Kaplan J. E., Benson C., Holmes K. K., Brooks J. T., Pau A., Masur H. (2009). Guidelines for prevention and treatment of opportunistic infections in HIV-infected adults and adolescents. *Morbidity and Mortality Weekly Report*.

[24] Sloan D. J., Parris V. (2014). Cryptococcal meningitis: epidemiology and therapeutic options. *Clinical Epidemiology*.

[25] Park B. J., Wannemuehler K. A., Marston B. J., Govender N., Pappas P. G., Chiller T. M. (2009). Estimation of the current global burden of cryptococcal meningitis among persons living with HIV/AIDS. *AIDS*.

[26] Vinnard C., MacGregor R. R. (2009). Tuberculous meningitis in HIV-infected individuals. *Current HIV/AIDS Reports*.

[27] Wall E. C., Cartwright K., Scarborough M. (2013). High mortality amongst adolescents and adults with bacterial meningitis in Sub-Saharan Africa: an analysis of 715 cases from Malawi. *PLoS ONE*.

[28] Veltman J. A., Bristow C. C., Klausner J. D. (2014). Meningitis in HIV-positive patients in sub-Saharan Africa: a review. *Journal of the International AIDS Society*.

[29] Domingo P., Pomar V., de Benito N., Coll P. (2013). The spectrum of acute bacterial meningitis in elderly patients. *BMC Infectious Diseases*.

[30] Chadwick D. R. (2005). Viral meningitis. *British Medical Bulletin*.

[31] Momméja-Marin H., Lafaurie M., Scieux C., Galicier L., Oksenhendler E., Molina J.-M. (2003). Herpes simplex virus type 2 as a cause of severe meningitis in immunocompromised adults. *Clinical Infectious Diseases*.

[32] Benjamin L. A., Kelly M., Cohen D. (2013). Detection of herpes viruses in the cerebrospinal fluid of adults with suspected viral meningitis in Malawi. *Infection*.

